# Brief report: harmonic analysis of the 30 Hz flicker ERG in early-stage diabetic retinopathy

**DOI:** 10.1007/s10633-025-10030-5

**Published:** 2025-05-31

**Authors:** J. Jason McAnany, Jason C. Park

**Affiliations:** 1 Department of Ophthalmology and Visual Sciences, University of Illinois at Chicago, 1855 W. Taylor St., MC/648, Chicago, IL USA; 2 Department of Biomedical Engineering, University of Illinois at Chicago, 851 South Morgan St., Chicago, IL 60607 USA

**Keywords:** Electroretinogram, Flicker, Diabetes, Diabetic retinopathy, Signal analysis

## Abstract

**Purpose:**

To determine if harmonic components of the 30 Hz flicker ERG are useful for detecting neural dysfunction in diabetics who have mild or no non-proliferative diabetic retinopathy (NPDR).

**Methods:**

Previously reported light-adapted flicker ERG data recorded from 20 diabetics who had no clinically-apparent retinopathy (NDR), 20 who had mild NPDR (MDR), and 20 non-diabetic controls were reanalyzed. From this dataset, the amplitude and phase of the 31.25 Hz flicker ERG fundamental and second harmonic were extracted. The 62.5 Hz flicker ERG fundamental was also extracted. Similar responses were also acquired prospectively from 10 controls, 5 NDR, and 5 MDR subjects, comprising a second dataset.

**Results:**

Analysis of variance indicated that both diabetic groups had normal amplitudes elicited by the 31.25 Hz stimulus (fundamental and second harmonic), whereas the 62.5 Hz amplitude was reduced significantly in both diabetic groups. This pattern was found in both the retrospective and prospective analyses.

**Conclusions:**

The second harmonic of the 31.25 Hz flicker response (equivalent to 62.5 Hz) was normal in early-stage DR, whereas the response to 62.5 Hz flicker stimuli was abnormal. The second harmonic of the ISCEV standard 30 Hz flicker ERG does not appear to be a useful indicator of neural dysfunction in early DR.

## Introduction

Diabetic retinopathy (DR) is the most severe ocular complication of diabetes and represents the leading cause of blindness among working-age adults [1]. In addition to the traditional association with abnormalities of the retinal vasculature [2, 3], there is also clear evidence of retinal neurodegeneration that can be apparent even in the earliest disease stages [4, 5]. Neural abnormalities of the retina have been documented in diabetics using objective, electrophysiological measures of the full-field flash ERG [6, 7]. The full-field flicker ERG recorded at the ISCEV standard flicker rate of approximately 30 Hz can also be abnormal in advanced DR [8, 9]. In individuals who are diabetic with no DR (NDR) or have mild NPDR (MDR), the 30 Hz flicker ERG is generally normal or only mildly attenuated, indicating that it may not provide an optimal approach for identifying DR-associated neural dysfunction in the earliest disease stages [6, 10, 11].

The flicker ERG has been recorded at flicker frequencies higher than 30 Hz in an effort to increase sensitivity for detecting neural dysfunction in early DR [6]. These studies suggest that the high frequency flicker ERG (elicited at flicker rates above approximately 55 Hz) can be abnormal in individuals who have NDR or MDR and normal flicker ERGs at the ISCEV standard 30 Hz flicker rate [6, 10]. Despite the potential usefulness of high frequency flicker for assessing retinal dysfunction in diabetes and other ocular disease, nearly all clinical and research studies record the flicker ERG at approximately 30 Hz, conforming to the ISCEV standard [12].

Specifically, ISCEV recommends the use of a 30 Hz stimulus flicker rate and analysis of the amplitude and timing of the response occurring at 30 Hz [12]. In addition to these standard time-domain analyses, the flicker ERG can be analyzed in the frequency domain. For these analyses, the response frequency that corresponds to the stimulus frequency is referred to as the fundamental (F) response component. In addition to the F response component, there are non-linear response components in the flicker ERG that occur at multiples of the stimulus frequency. That is, a 30 Hz stimulus will elicit a response that occurs at 30 Hz (F) and at multiples of the stimulus frequency, termed “harmonics” (e.g., 60 Hz, 90 Hz, 120 Hz). It is possible that the second harmonic of the ISCEV standard 30 Hz response, equivalent to 60 Hz, might be useful for approximating the amplitude attenuation observed in response to high frequency stimulation. This would be of clinical value because sensitivity for detecting retinal dysfunction in DR using the ISCEV standard 30 Hz ERG might be increased by examining the harmonics rather than the fundamental.

In contrast to this expectation, however, a previous analysis showed that the F elicited by 16 Hz flicker was normal in DR, as were the high frequency harmonic components including 2F (32 Hz), 3F (48 Hz), and 4F (64 Hz) [13]. The harmonic components elicited at the ISCEV standard 30 Hz flicker rate have not reported in DR subjects and the neural generators of the 16 and 30 Hz flicker ERG differ. For example, the direct cone photoreceptor contribution at 16 Hz is larger than at 30 Hz [14]. Thus, it is presently unclear whether or not DR might affect the harmonic components of the 30 Hz flicker ERG.

The purpose of this brief report was to reanalyze previously published flicker ERG data to determine if the 2F of the 30 Hz flicker ERG is abnormal in early DR, and therefore a useful marker of neural dysfunction. In addition, new flicker ERG data were acquired from a small sample of early-stage DR subjects elicited by 31.5 and 63 Hz stimuli using the RetEval handheld ERG instrument. Patterns of abnormality observed with the RetEval are compared to the previously published data obtained with conventional ganzfeld stimulation.

## Methods

The flicker ERG data were obtained from two sources: 1) retrospective data were obtained from a previous study of the flicker ERG in diabetes [13]; 2) prospective data were obtained from 20 subjects, including 10 visually-normal controls, 5 diabetics who had NDR, and 5 with MDR. The study was performed in accordance with the ethical standards established in the 1964 Declaration of Helsinki and its later amendments, institutional review board approval was obtained at the University of Illinois Chicago, and the experiments were undertaken with the understanding and written consent of each subject.

### Retrospective study; subjects, apparatus, stimuli, and analysis

As described in the original report [13], the sample consisted of 40 individuals diagnosed with type-2 diabetes (mean age ± SD: 52.8 ± 8.3 years) and 20 visually-normal, non-diabetic control subjects (mean age ± SD: 51.9 ± 12.2 years). Each subject was examined by a retina specialist and clinically classified as NDR (N = 20) or MDR (N = 20).

The pupil of the tested eye was dilated with 2.5% phenylephrine hydrochloride and 1% tropicamide drops. ERGs were recorded monocularly using DTL electrodes with the fellow eye patched. Gold-cup electrodes were used as reference (ear) and ground (forehead). Sinusoidal flicker stimuli were generated by and presented in a ColorDome desktop ganzfeld system (Diagnosys LLC, Lowell, MA). A uniform field consisting of 100 cd/m^2^ red (632 nm) and 100 cd/m^2^ green (516 nm) light was modulated sinusoidally (100% contrast) at temporal frequencies of 31.25 Hz and 62.5 Hz. Each flicker train had a duration of 1,024 ms. Prior to the recordings, the subject adapted for 2 min to a uniform field consisting of 100 cd/m^2^ 632 nm and 100 cd/m^2^ 516 nm light. A minimum of 5 responses for each temporal frequency were obtained and averaged for analysis. The responses were band pass filtered (0.30 – 300 Hz) and processed using custom scripts programmed in MatLab to derive the amplitude and phase of the fundamental and harmonics by Fourier analysis. In addition to these amplitude and phase measurements, the amplitude of noise in the response was also quantified. Specifically, noise amplitude was defined as the amplitude at frequencies in the Fourier spectrum that neighbor the stimulus (or harmonic) frequency, consistent with previous definitions [15]. Statistical analyses were performed in SigmaPlot (version 12; SYSTAT, Chicago, IL).

### Prospective study; subjects, apparatus, stimuli, and analysis

Ten individuals diagnosed with type-2 diabetes were recruited from the Department of Ophthalmology and Visual Sciences at the University of Illinois Chicago. Each subject underwent a fundus exam performed by a retina specialist and the subjects were clinically classified as NDR (N = 5; mean ± SD age 53.0 ± 10.2 years) or MDR (N = 5; mean ± SD age 53.4 ± 5.3 years) according to the early treatment of diabetic retinopathy study (ETDRS) scale [2]. Other than diabetes, no subject had systemic disease known to affect retinal function, ocular disease, or significant cataract. No subject had a history of treatment or diabetic macular edema at the time of testing. In addition, 10 visually-normal, non-diabetic control subjects were also recruited (mean age ± SD: 50.5 ± 10.23 years). Mean age of the control and diabetic subjects did not differ significantly (F = 0.28, *p* = 0.76).

Flicker ERGs were obtained monocularly with the RetEval device (LKC Technologies Inc., Gaithersburg, MD). The test was performed in a dark room with the fellow eye unoccluded. The stimuli consisted of sinusoidally modulated 31.5 Hz and 63.0 Hz flickering light generated by green (530 nm) and blue (470 nm) LEDs (CIE: 0.150, 0.339). Stimulus mean retinal illuminance (10,000 Td) was maintained consistently throughout presentation and was equal for the 31.5 and 63.0 Hz stimuli. This was achieved by monitoring the subject’s pupil size using a built-in infrared camera and adjusting the time-integrated luminance. The Michelson contrast of the sinewave stimuli was 100%. A single sweep was obtained at each frequency that ranged in duration from approximately 5 to 15 s. The sweep duration depended on the standard error of the fundamental flicker timing. In cases where the fundamental response time had a high standard error (i.e., variable/noisy), the stimulus could last up to 15 s. No pre-adaptation to the stimulus was provided.

ERGs were recorded with DTL electrodes (Diagnosys, LLC); gold-cup electrodes were used as reference (ear) and ground (forehead). The pupils were not pharmacologically dilated, given the ability to achieve constant retinal illuminance as noted above. The amplifier bandpass was DC to 512 Hz and the sampling frequency was 1,953 Hz. The flicker ERG waveforms were exported and processed using Fourier analysis implemented in MatLab to derive the amplitude and phase of the fundamental and harmonic responses. Statistical analyses were performed in SigmaPlot (version 12; SYSTAT, Chicago, IL).

## Results

### Retrospective data

**Fig. 1:**
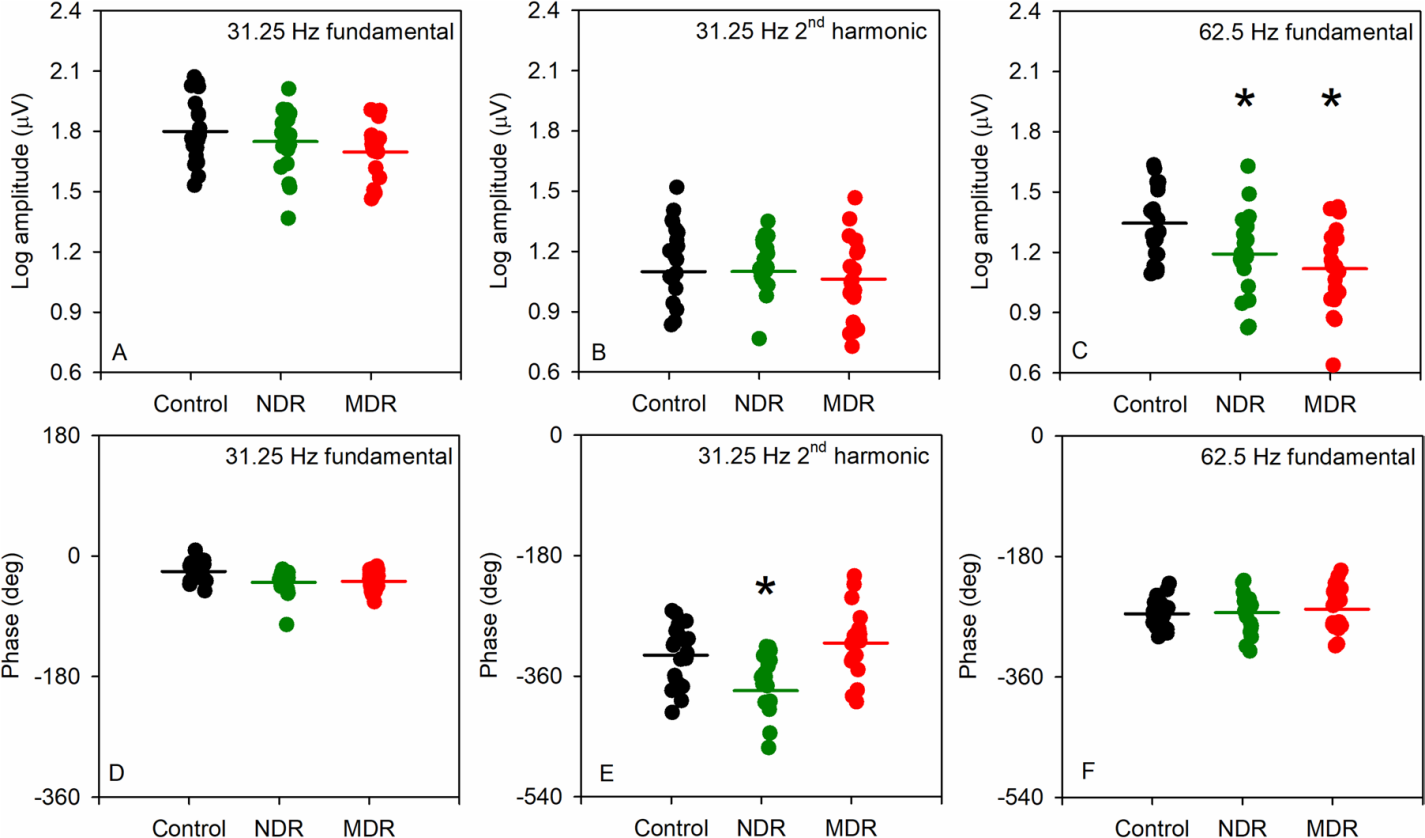
Retrospective analysis of flicker ERG amplitude (**A**–**C**) and phase (**D**–**F**). Log fundamental amplitude for the 31.25 Hz stimulus (**A**), second harmonic amplitude for the 31.25 Hz stimulus (**B**), and fundamental amplitude for the 62.5 Hz stimulus (**C**) are shown. Fundamental phase for the 31.25 Hz stimulus (**D**), second harmonic phase for the 31.25 Hz stimulus (**E**), and fundamental phase for the 62.5 Hz stimulus (**E**) are shown in the second row. Controls are indicated by the black circles, NDR subjects by green circles, and MDR subjects by red circles. The horizontal bars mark the group means. Asterisks mark statistically significant differences from the control group (*p* < 0.05)

Figure 1 shows the log fundamental amplitude elicited by the 31.25 Hz stimulus (A), the log second harmonic amplitude elicited by the 31.25 Hz stimulus (B), and the log fundamental amplitude elicited by the 62.5 Hz stimulus (C). Each circle represents an individual control (black), NDR (green), or MDR (red) subject. The 31.25 Hz response group means (horizontal bars) were similar for the fundamental and second harmonic. By contrast, the mean response amplitude for the 62.5 Hz stimulus was reduced for the NDR and MDR subjects compared to the controls. For all subject groups, the mean noise amplitude, as defined in the Methods, was less than 0.23, 0.12, and − 0.03 log µV for the 31.25 Hz fundamental, 31.25 Hz second harmonic, and 62.5 Hz fundamental responses, respectively. A repeated measures two-way analysis of variance (ANOVA), with main effects of group (control, NDR, MDR) and response component (31.25 Hz fundamental, 31.25 Hz second harmonic, 62.5 Hz fundamental), was performed to compare the amplitudes among the groups. There was no significant effect of group (F = 2.51, *p* = 0.09), but there was a significant effect of component (F = 453.24, *p* < 0.001), as well as a significant interaction between these main effects (F = 3.26, *p* = 0.01). For the 31.25 Hz fundamental response, Holm-Sidak pairwise comparisons indicated no statistically significant amplitude difference between the controls and either diabetic group (both t < 1.61, *p* > 0.21). Similarly, for the 31.25 Hz second harmonic, Holm-Sidak pairwise comparisons indicated no statistically significant amplitude difference between the controls and either diabetic group (both t < 0.57, *p* > 0.81). For the 62.5 Hz fundamental, Holm-Sidak pairwise comparisons indicated statistically significant amplitude differences between the controls and the NDR subjects (t = 2.42, *p* = 0.02) and between the controls and the MDR subjects (t = 3.57, *p* = 0.001).

Figure 1 (second row) shows the fundamental phase elicited by the 31.25 Hz stimulus (D), the second harmonic phase elicited by the 31.25 Hz stimulus (E), and the fundamental phase elicited by the 62.5 Hz stimulus (F). The 31.25 Hz fundamental response phases were similar for the three groups. The group means for the second harmonic phase of the 31.25 Hz stimulus differed somewhat among the groups and there was considerable variation within groups. The mean response phase for the 62.5 Hz stimulus was similar among the groups. Repeated measures two-way ANOVA indicated a significant effect of group (F = 3.93, *p* = 0.03), component (F = 2,299.43, *p* < 0.001), as well as a significant interaction between these main effects (F = 7.81, *p* < 0.01). For the 31.25 Hz fundamental, Holm-Sidak pairwise comparisons indicated no statistically significant phase difference between the control group and either diabetic group (both t < 1.60, *p* > 0.21). However, for the 31.25 Hz second harmonic, there was a small, but statistically significant, phase difference between the controls and the NDR subjects (t = 3.58, *p* < 0.001), but not between the controls and the MDR subjects (t = 1.77, *p* = 0.08). For the 62.5 Hz fundamental, there was no statistically significant phase difference between the controls and either diabetic group (both t < 0.68, *p* > 0.74).

### Prospective data

**Fig. 2:**
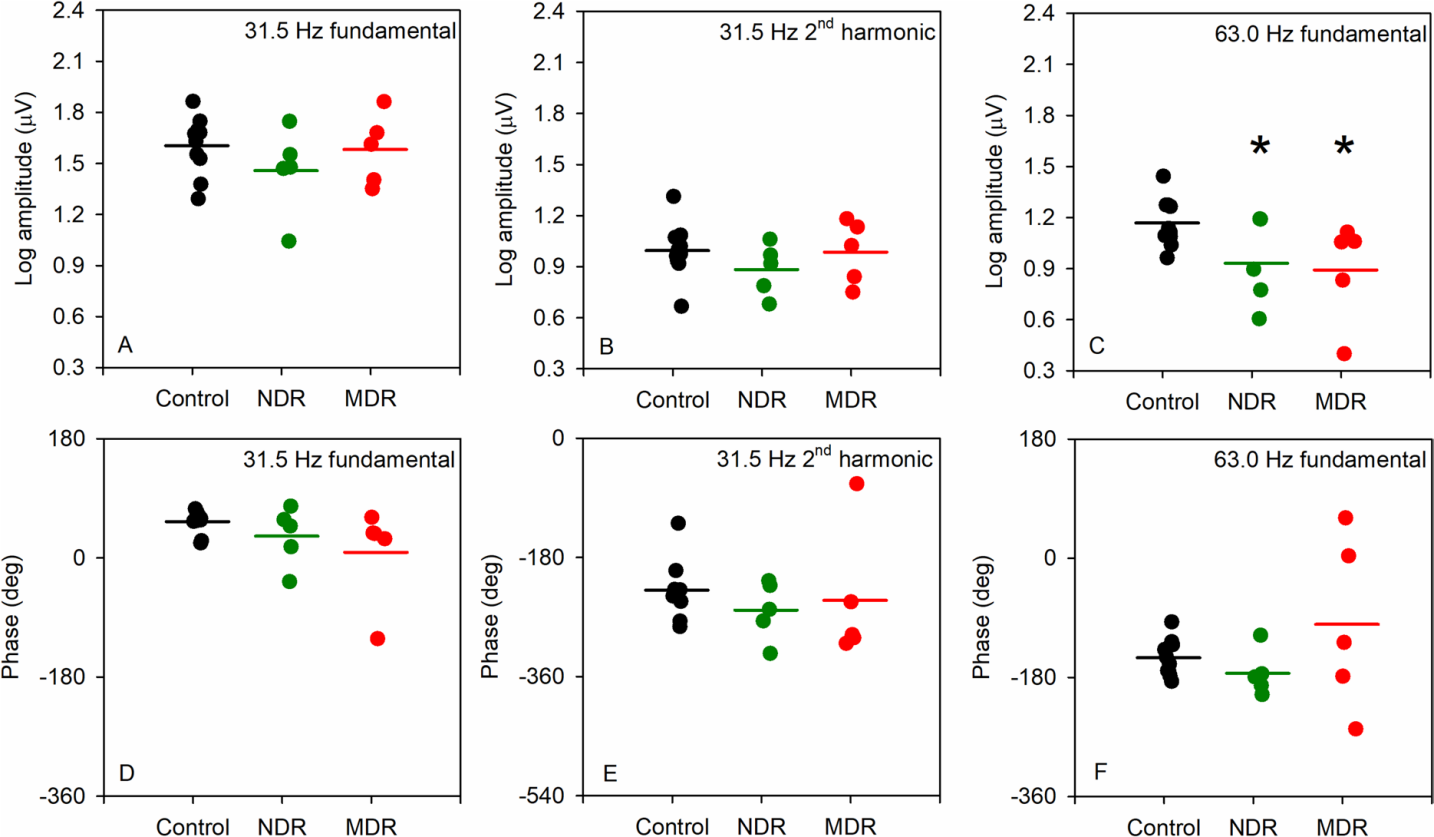
Prospective analysis of flicker ERG amplitude (**A**–**C**) and phase (**D**–**F**). Conventions are as in Fig. 1

Figure 2 shows the log amplitudes for the three groups for each component obtained with the RetEval in the same format as Fig. 1. As for the retrospective data, the 31.5 Hz amplitude group means were similar for the fundamental (Fig. 1A) and second harmonic (Fig. 1B), whereas the mean response amplitude for the 63 Hz stimulus was reduced for the NDR and MDR subjects compared to the controls (Fig. 1C). For all subject groups, the mean noise amplitude, as defined in the Methods, was less than 0.32, 0.08, and 0.24 log µV for the 31.5 Hz fundamental, 31.5 Hz second harmonic, and 63 Hz fundamental responses, respectively. A repeated measures two-way ANOVA, found no significant effect of group (F = 1.54, *p* = 0.24), but there was a significant effect of component (F = 208.94, *p* < 0.001), as well as a significant interaction between these main effects (F = 4.09, *p* = 0.008). For the 31.5 Hz fundamental response, Holm-Sidak pairwise comparisons indicated no statistically significant amplitude differences between the controls and either diabetic group (both t < 1.36, *p* > 0.34). Similarly, for the 31.5 Hz second harmonic, Holm-Sidak pairwise comparisons indicated no statistically significant amplitude differences between the controls and either diabetic group (both t < 1.04, *p* > 0.52). For the 63 Hz fundamental, Holm-Sidak pairwise comparisons indicated statistically significant amplitude differences between the control and NDR groups (t = 2.21, *p* = 0.04) and between the control and the MDR groups (t = 2.57, *p* = 0.03).

Figure 2 (second row) shows the fundamental phase elicited by the 31.5 Hz stimulus (D), the second harmonic phase elicited by the 31.5 Hz stimulus (E), and the fundamental phase elicited by the 63 Hz stimulus (F). The 31.5 Hz fundamental phase was generally similar among the three groups, which was also the case for the second harmonic of the 31.5 Hz stimulus and for the 63 Hz fundamental. Repeated measures two-way ANOVA indicated no significant effect of group (F = 1.30, *p* = 0.30), and the interaction between group and component was also not significant (F = 1.16, *p* = 0.35).

## Discussion

The purpose of this brief report was to determine if the second harmonic of the 30 Hz flicker ERG could serve as a useful marker of neural dysfunction in early stage DR. The two primary findings are: (1) the 30 Hz flicker ERG fundamental and second harmonic components were generally normal in NDR and MDR subjects; (2) the 60 Hz flicker ERG fundamental was significantly reduced in NDR and MDR groups. This pattern of findings is consistent in both the retrospective and prospective datasets and indicates that the second harmonic of 30 Hz (60 Hz) is not equivalent to the fundamental response elicited by 60 Hz flicker. Although the 60 Hz flicker ERG may be useful for studying neural dysfunction in diabetes, it is important to note that this stimulus is not included in the ISCEV full-field ERG standard [12]. Furthermore, the response to 60 Hz flicker is considerably smaller than the response to 30 Hz flicker, which may create challenges for conventional peak-to-trough time-domain analyses of the response. Nevertheless, all subjects had a 60 Hz flicker response that statistically exceeded the noise level [15].

The second harmonic of the 31.25 Hz flicker response was slightly delayed (36° on average) for the NDR subjects when measured with the ColorDome. However, only two of the NDR subjects had a phase value outside of the normal range and the phase delay was not significant for the MDR subjects. Neither subject group had a phase delay for any response component measured with the RetEval, but the small sample size for this preliminary study was not appropriately powered to detect small differences among groups. Given the relatively small phase difference between the NDR and control subjects and that the effect was not observed in the more severely affected MDR subjects, the phase of the second harmonic is unlikely to be a useful marker of neural dysfunction in diabetes. However, larger samples of subjects with a broader range of disease severity are needed to fully evaluate this conclusion.

**Fig. 3:**
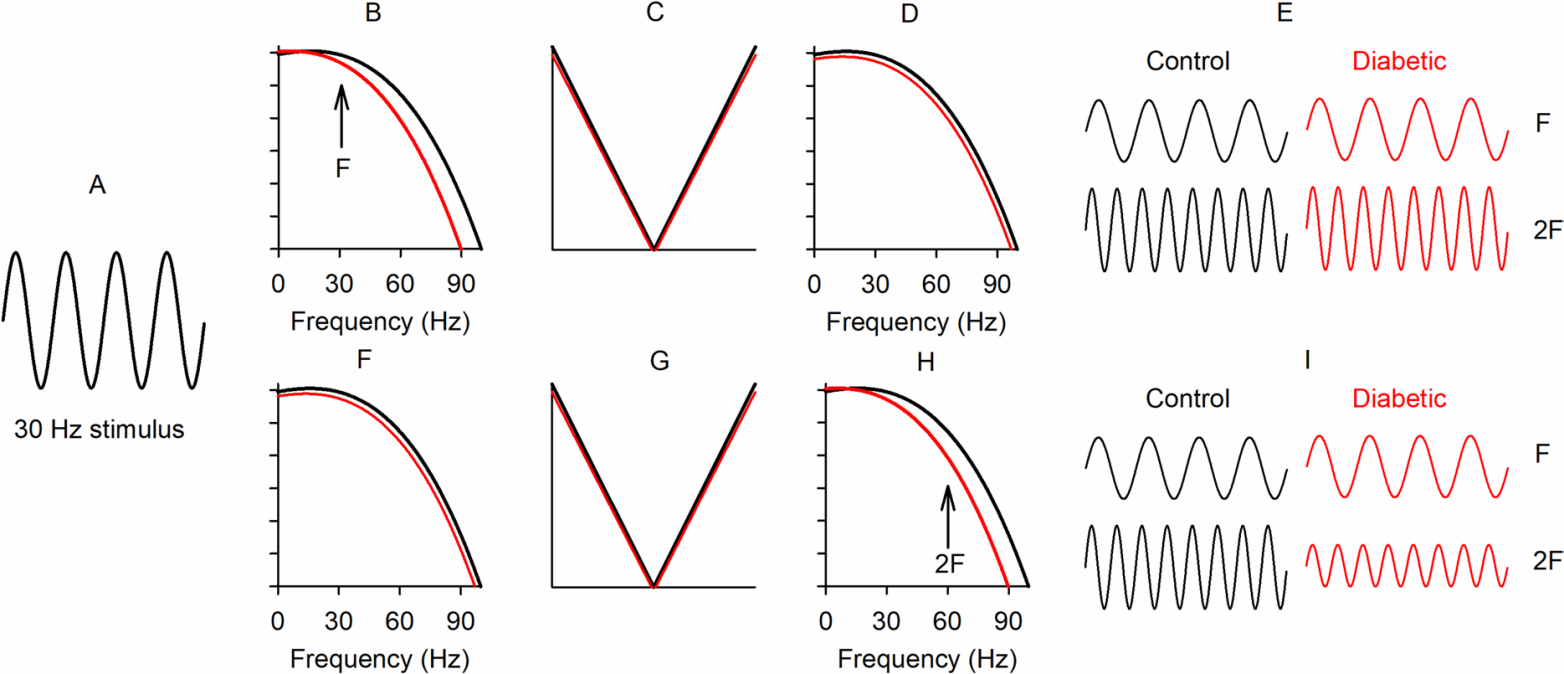
Schematic illustration of the sandwich model of the flicker ERG. A 30-Hz sinewave stimulus is shown in A. The top row (**B**–**E**) shows the effects of an early retinal abnormality at the level of the first low-pass filter. The bottom row (**F**–**I**) shows the effects of a later retinal abnormality at the level of the second low-pass filter. Black filters and waveforms represent controls, whereas red filters and waveforms represent diabetic subjects. If the abnormality is only at the first linear filter, the 30 Hz fundamental and harmonics will be normal (top). If the abnormality is only at the second linear filter, the 30 Hz fundamental will be normal, but the harmonics will be abnormal (bottom)

The explanation for why the second harmonic amplitude of the 30 Hz flicker ERG is normal in early DR, whereas the ERG amplitude elicited by 60 Hz flicker is abnormal can be understood in the context of a previously proposed “sandwich model” [16, 17] of the generation of the flicker ERG. This model posits that a retinal nonlinearity is sandwiched between two linear filters, as schematized in Fig. 3. This produces a linear response component that occurs at the stimulus frequency (F) and nonlinear response components that occur at multiples of the stimulus frequency (harmonics). Based on this model, the site of dysfunction in diabetes has been proposed to occur at the first linear filter [13]. As shown in Fig. 3, the response to a 30-Hz sinewave stimulus (A) from the diabetic retina is passed through a linear filter (Fig. 3B; red) without substantial attenuation compared to a non-diabetic retina (Fig. 3B; black). By contrast, the response to a 60 Hz stimulus would be attenuated by the first linear filter in the diabetic retina (Fig. 3B; red). The normal 30-Hz response from the diabetic retina is then passed through a nonlinearity that generates the harmonics (Fig. 3C), which would also be normal in the diabetic retina. Finally, the fundamental and harmonics are passed through a second linear filter (Fig. 3D), which is also assumed to be normal in the diabetic retina. Thus, if the site of dysfunction is early in the retina (at the first linear filter), the 30 Hz response and the harmonics will be normal (Fig. 3E), whereas the 60 Hz response and the harmonics will be attenuated (not shown). The bottom row of Fig. 3 provides an alternative hypotheses and illustrates the effects on the fundamental and harmonics if the site of dysfunction occurs at the second linear filter. In this case, the response to a 30 sinewave stimulus (3A) is not attenuated by the diabetic retina (red; Fig. 3F). The normal fundamental response is passed through the nonlinearity, which generates normal harmonics in the diabetic retina (Fig. 3G). Finally, the fundamental (30 Hz) and second harmonic (60 Hz) are passed through the second linear filter (Fig. 3H), which attenuates the second harmonic but not the fundamental (Fig. 3I). Thus, if the site of dysfunction is at the first linear filter, the 30 Hz response and the harmonics will be normal (3E), as observed in the subject data of Figs. 1 and 2. Conversely, if the site of dysfunction is at the second linear filter, the 30 Hz response will be normal and the harmonics will be attenuated; this was not observed in the data of Figs. 1 and 2. In summary, an early site of damage (first linear filter) would account for why the second harmonic of the 30 Hz flicker ERG (equivalent to 60 Hz) is normal in DR, whereas the ERG elicited by 60 Hz flicker is abnormal.

In conclusion, the high frequency flicker ERG (e.g., 60 Hz) is useful for evaluating neural dysfunction in diabetics who have NDR or MDR, as this response can be significantly abnormal. The second harmonic of the 30 Hz flicker ERG is not useful in this regard. Taken together, previous findings and the current data suggest that the use of higher flicker rates than traditionally employed in clinical ERG recordings could be useful for identifying and understanding temporal processing abnormalities in DR.

## Declarations

### Statement on the welfare of animals

No animal experiments were performed.
